# International Dispersal of Dengue through Air Travel: Importation Risk for Europe

**DOI:** 10.1371/journal.pntd.0003278

**Published:** 2014-12-04

**Authors:** Jan C. Semenza, Bertrand Sudre, Jennifer Miniota, Massimiliano Rossi, Wei Hu, David Kossowsky, Jonathan E. Suk, Wim Van Bortel, Kamran Khan

**Affiliations:** 1 European Centre for Disease Prevention and Control (ECDC), Stockholm, Sweden; 2 Division of Infectious Diseases, St. Michael's Hospital, Toronto, Ontario, Canada; 3 Faculty of Medicine, University of Toronto, Toronto, Ontario, Canada; U.S. Naval Medical Research Unit Six, United States of America

## Abstract

**Background:**

The worldwide distribution of dengue is expanding, in part due to globalized traffic and trade. *Aedes albopictus* is a competent vector for dengue viruses (DENV) and is now established in numerous regions of Europe. Viremic travellers arriving in Europe from dengue-affected areas of the world can become catalysts of local outbreaks in Europe. Local dengue transmission in Europe is extremely rare, and the last outbreak occurred in 1927–28 in Greece. However, autochthonous transmission was reported from France in September 2010, and from Croatia between August and October 2010.

**Methodology:**

We compiled data on areas affected by dengue in 2010 from web resources and surveillance reports, and collected national dengue importation data. We developed a hierarchical regression model to quantify the relationship between the number of reported dengue cases imported into Europe and the volume of airline travellers arriving from dengue-affected areas internationally.

**Principal Findings:**

In 2010, over 5.8 million airline travellers entered Europe from dengue-affected areas worldwide, of which 703,396 arrived at 36 airports situated in areas where *Ae. albopictus* has been recorded. The adjusted incidence rate ratio for imported dengue into European countries was 1.09 (95% CI: 1.01–1.17) for every increase of 10,000 travellers; in August, September, and October the rate ratios were 1.70 (95%CI: 1.23–2.35), 1.46 (95%CI: 1.02–2.10), and 1.35 (95%CI: 1.01–1.81), respectively. Two Italian cities where the vector is present received over 50% of all travellers from dengue-affected areas, yet with the continuing vector expansion more cities will be implicated in the future. In fact, 38% more travellers arrived in 2013 into those parts of Europe where *Ae. albopictus* has recently been introduced, compared to 2010.

**Conclusions:**

The highest risk of dengue importation in 2010 was restricted to three months and can be ranked according to arriving traveller volume from dengue-affected areas into cities where the vector is present. The presence of the vector is a necessary, but not sufficient, prerequisite for DENV onward transmission, which depends on a number of additional factors. However, our empirical model can provide spatio-temporal elements to public health interventions.

## Author Summary

The global disease burden of dengue is staggering. Continuous expansion and vaccine failures illustrate the limitations of current dengue control efforts. Novel approaches and additional tools are required to combat and contain the disease. In Europe, dengue infections are rare and the last outbreak of dengue occurred in the late 1920s, in Greece. In 2010, however, local transmission occurred in France and Croatia. Based on 2010 data, we present a novel quantitative model of the risk of dengue importation for Europe. The 2010 model predicts the risk of dengue importation to be greatest for Milan, Rome and Barcelona in August, September and October, precisely when vector activity is the highest. With the current expansion of the vector in Europe, more cities are projected to be at risk in the future. Thus, the model based on 2010 data quantifies the likelihood and timing of importation. This approach employs global travel data to assess dengue importation risk in the EU and illustrates how quantitative models could tailor infectious disease control to certain regions and time periods.

## Introduction

Dengue has emerged as the most important viral mosquito-borne disease globally, taking on pandemic proportions with a 30-fold increase in disease burden over the last half-century [1]–[4]. The global burden of dengue is difficult to estimate but ranges from 50 to 390 million infections per year [4], [5]. Similarly, the global geographic range is challenging to demarcate, but it includes up to 128 countries where the disease is now endemic or epidemic [4]–[6]. Transmission occurs predominantly in the tropical and sub-tropical regions of the world, threatening almost half of the world's population [4]. The lack of highly efficacious vaccines, antiviral therapies, therapeutic interventions, and efficient vector abatement strategies hampers dengue control efforts [3], [7].

*Aedes aegypti* is the predominant mosquito vector that transmits the dengue virus (four virus serotypes: DENV 1–4) to humans, whereas *Aedes albopictus* is also a competent but less effective vector [8]. Historically, these vectors have expanded their habitat due to globalized travel and trade. From West Africa, *Ae. aegypti* dispersed around the world and colonized Europe at the beginning of the 20^th^century, but has since receded. However, in 2005 *Ae. aegypti* was first reported in Madeira, Portugal and has subsequently expanded over the southern part of the island [9]. It is also present around the Black Sea coast in Russia, Abkhazia, and Georgia. *Ae. albopictus* is considered one of the most invasive mosquito species of public health importance in the world; it originated in Southeast Asia, and has rapidly expanded internationally over the last half-century. In Europe, it appeared first in Albania in 1979 and was subsequently introduced in Italy in used tires in the 1990s from where it most likely spread to other Mediterranean countries [10], [11]. *Ae. albopictus* is now present in at least 15 countries (either established or recently recorded) and continues to progressively expand. Entomological monitoring activities in the Mediterranean indicate that the development period for *Ae. albopictus* starts in April and tapers off in October/November with activity peaks in June/July–September [12]–[14]. However, most monitoring is conducted with ovitraps and not actual counts of adult mosquito densities. Nonetheless, the number of female mosquitoes per hectare (female mosquito density) has been correlated with the mean egg density of the week after sampling [15]. Thus, the *Ae. albopictus* activity peak in this Mediterranean region are the summer months, based on entomological data from France, Greece, and Croatia [12]–[14].

For many Europeans, dengue endemic areas such as the Caribbean Islands and Southeast Asia are popular travel destinations [16]. The majority of these travellers are vacationers and business travellers but also visitors of friends and family [17]. Any returning traveller from the tropics or subtropics, even if the region has not been classified as endemic, should be considered at risk for DENV infection [18]. Rapid international air travel permits infected travellers to arrive in Europe during their viremic period, which can last up to 5 days post-onset of illness. During this time naïve invasive *Aedes* mosquitoes that are now present in several European countries may bite a viremic traveller and become infected [19]. Infected mosquitos can then in turn transmit DENV to a susceptible individual locally. Exposure of viremic individuals and susceptible individuals to competent mosquito vectors could spawn local outbreaks.

In fact, autochthonous transmission has recently occurred in areas where *Aedes* mosquitoes are established [20], [21]. In September 2010, two cases of dengue with no recent history of international travel or blood transfusion were identified in the Mediterranean coast of France [20]; similarly, two cases were reported along the Adriatic coast in Croatia along with 15 additional individuals between August and October with an indication of recent DENV infection [21]. This evidence strongly suggests that local transmission occurred in Europe for the first time in many decades. Consequently, the environmental/climatic envelope is permissive for dengue outbreaks in the Mediterranean area of Europe [22]. In September 2012, an outbreak of more than 2000 dengue cases occurred in Madeira, Portugal in areas where *Ae. aegypti* is known to exist [9]. The island is well-connected with scheduled flights to tropical countries where dengue is endemic; three imported cases from Angola and Brazil were identified during the outbreak while 78 DENV-infected travellers (presumably mainly tourists) have returned from Madeira to the European mainland.

Thus, Europe is at risk for dengue importation and subsequent re-emergence, based on five important developments: 1) dengue incidence has significantly increased globally over the past few decades; 2) *Ae. albopictus*, a competent vector for dengue, has established itself in many southern European countries and is rapidly expanding; 3) international air travel into Europe from dengue-affected areas of the world has augmented, creating myriad opportunities for viremic travellers to encounter receptive invasive *Aedes* mosquitoes in Europe; 4) environmental/climatic conditions in Europe are permissive for a local cycle of transmission, as the recent outbreaks have shown; 5) infected mosquitoes from dengue-affected areas themselves can be transported through air traffic and introduced into Europe where the climatic environments are permissive.

These developments require a quantitative approach for anticipating the risk of dengue outbreaks in receptive areas, taking into consideration factors such as the worldwide burden and seasonality of dengue, the magnitude and seasonal pattern of travellers arriving from dengue-affected areas, and the seasonality and distribution of competent mosquito populations within Europe. We present a model based on 2010 data that relates air travellers from dengue affected areas to dengue importation to Europe. Such models can be applied to other settings and time periods and can support integrated surveillance of human cases and vectors [23]–[25].

## Methods

### Data sources

Worldwide dengue outbreak notifications were compiled from DengueMap and the Communicable Disease Threat Report (CDTR) produced by ECDC [26], [27]. The CDTRs are weekly reports generated by the Epidemic Intelligence team at ECDC regarding communicable disease threats and contains a dedicated section on dengue. Rather than gauging qualitative dengue prevalence/risk [5], [6] our assessment is based on dengue events picked up by web searches and from confidential/official sources such as Early Warning and Response System (EWRS); Program for Monitoring Emerging Diseases (ProMED); Medical Information System (MediSys); Global Public Health Intelligence Network (GPHIN); etc. Weekly notifications from these sources of worldwide locations with dengue activity were evaluated for the year 2010 and geocoded by month. We mapped the passenger volume of outbound flights to Europe from these dengue active areas (endemic and epidemic) at the city level by month and aggregated by quarter of 2010. The year 2010 was chosen because the corresponding country-level importation data were only available for that year (see below). “Active airports” were defined as those within a 200 km perimeter of a dengue outbreak for that month, as identified by DengueMap and/or CDTR.

The number of reported dengue importations was collected for the most recent year for which data were available (2010). Data from high air traffic countries (France, Germany, Italy, Spain, Sweden, Netherlands and United Kingdom) were obtained from national surveillance institutes, scientific publications and/or reference laboratories and aggregated by month [28]–[31]. We assumed that symptomatic European citizens and visitors were equally likely to be captured by national health care systems.

The volume of international travellers initiating trips by month in 2010 from dengue active airports worldwide with a final destination in Europe (i.e. accounting for all connecting flights) was calculated by analysing anonymized flight itinerary data obtained from the International Air Transport Association (IATA). All travellers on commercial flights, including scheduled charters, were captured. These passenger data represent approximately 93% of the world's commercial air traffic, while the remainder were estimated using market intelligence. The distribution of number of travellers arriving into Europe from active airports was than overlaid with European vector surveillance data collected by ECDC (Vbornet) for *Ae. albopictus* using ESRI ArcGIS [11]. We present maps with 2010 IATA data with both the 2010 and the 2013 *Ae. Albopictus* distribution.

### Statistical analysis

The number of dengue cases imported into European countries (dependent variable) was modelled using multilevel mixed-effects regression for count outcomes with the month of reporting and monthly volume of travellers from dengue-affected areas worldwide as independent variables (predictors). Considering the group structure of the data at the country level, a hierarchical count model random-intercept and random-coefficient model at country level was performed. After data exploration, a negative binomial distribution and log-link function was used in order to take into account over-dispersion of the outcome variable. Therefore, we performed a Generalized Linear Mixed Models by using gllamm with STATA 12 [32], [33]. Predictions, model diagnostic measures, residuals plots and detection of outliers were examined according to best practices of model post-estimation [34]. To assess the stability of the regression coefficient, a sensitivity analysis was carried out by removing outliers. Regression coefficients and Incidence Rate Ratio (IRR) with 95% confidence interval are reported. The analyses and graphics were conducted using R 3.0 and STATA 12.

## Results

**Figure 1:**
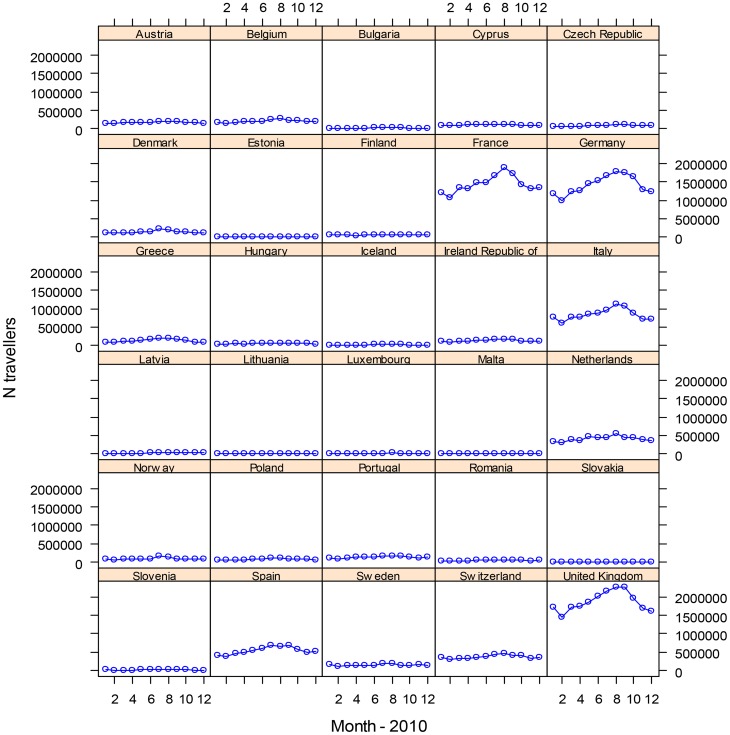
Number of international air travellers arriving in the EU, by country and month, 2010.

Over 103 million travellers entered Europe on commercial flights in 2010; the distribution by destination country and month is shown in figure 1. Of the total number of travellers, 26.6% originated from North Africa and West Asia, 25.0% from North America, 13.5% from East Asia, 4.9% from South Asia, 7.4% from Eastern Europe and Central Asia, 6.9% from Sub-Saharan Africa, 4.9% from South America, 4.5% from Mexico, Central America and the Caribbean, 4.1% from West, North and South Europe and around 2% from Australia, New Zealand and the Pacific Islands. The monthly average number of international travellers for the peak travel season (third quarter: July, August and September 2010) for selected high traffic countries was approximately 2.2 million for the United Kingdom (UK); 1.7 million for France; 1.7 million for Germany; 1 million for Italy; 669,000 for Spain; 484,000 for the Netherlands; and 183,000 for Sweden.

**Figure 2:**
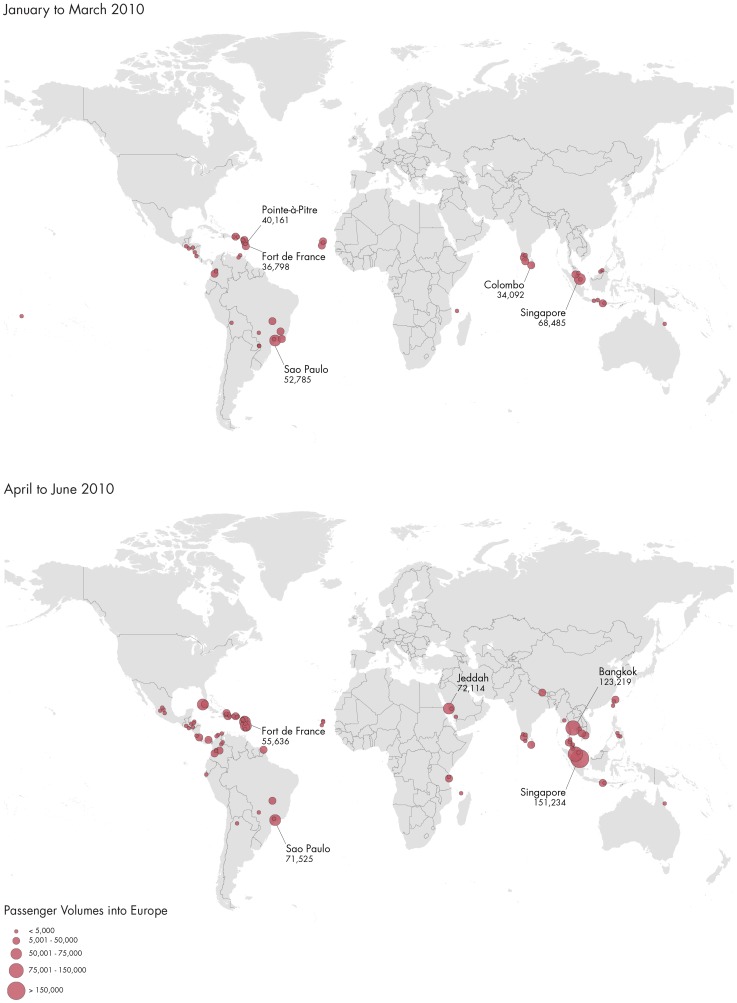
Passenger volume of outbound flights to Europe from dengue active areas (endemic and epidemic) at the city level by quarter, 2010.

**Figure 3:**
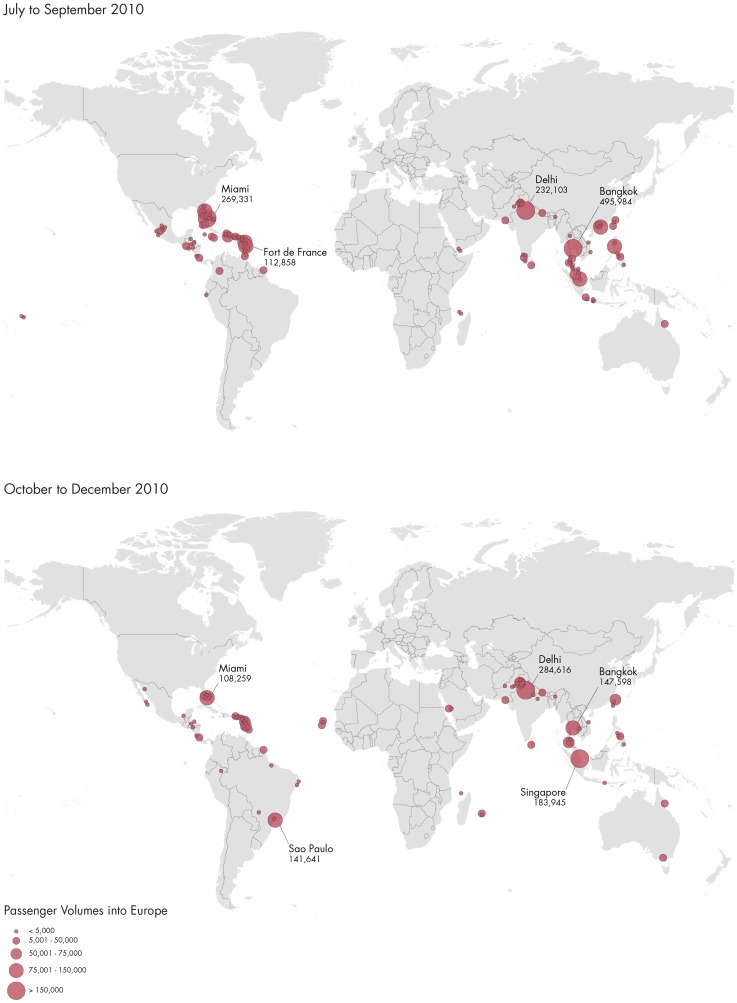
Passenger volume of outbound flights to Europe from dengue active areas (endemic and epidemic) at the city level by quarter, 2010.

**Figure 4:**
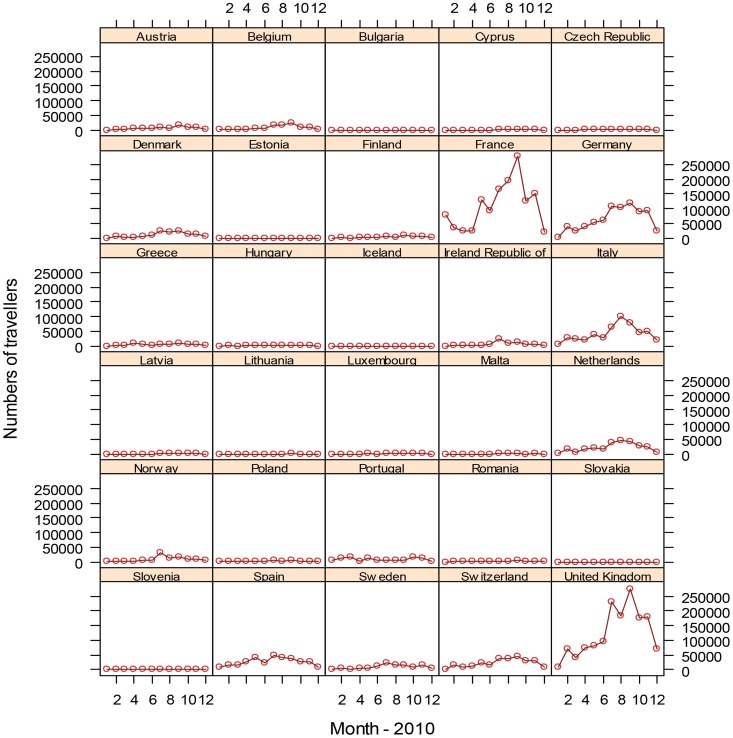
Number of international air travellers from dengue affected areas (endemic and epidemic) arriving in the EU, by country and month, 2010.

**Figure 5:**
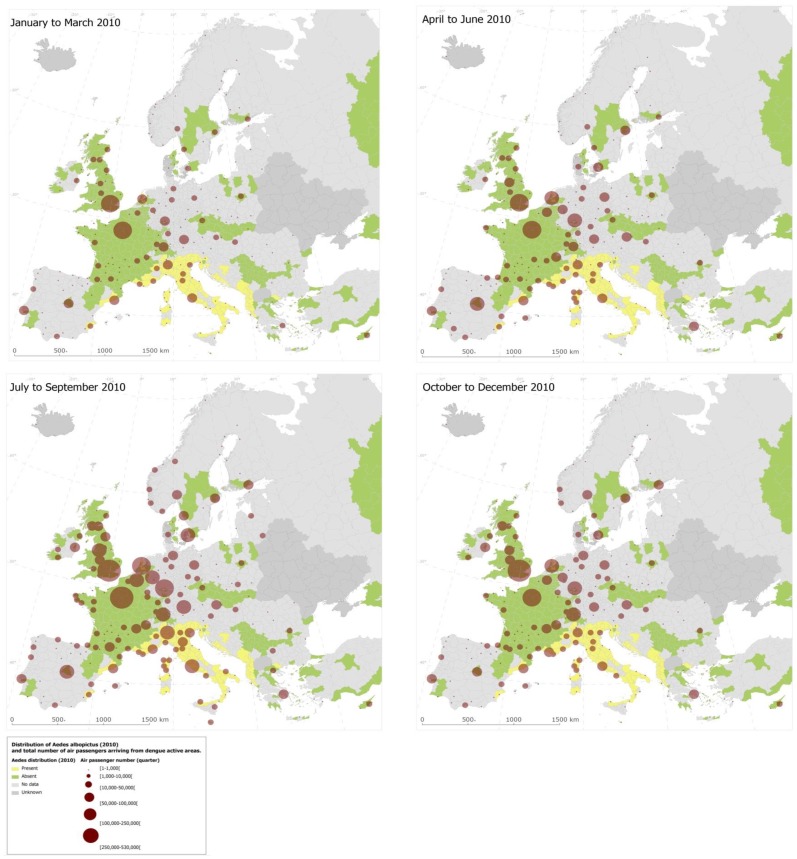
Airport-level final destination of international travellers from dengue affected areas (endemic and epidemic) by quarter for 2010, overlaid with the presence of *Ae. albopictus*, 2010.

More than 5.8 million travellers entered Europe from dengue-affected areas in 2010 (figure 2 and 3); country-level arrival by month is shown in figure 4. Of the total number of travellers from dengue-affected areas, 30.7% originated from East Asia, 13.8% from South Asia, 9.5% from North Africa and West Asia, 16.7% from Sub-Saharan Africa, 0.9% from Eastern Europe and Central Asia, 14.2% from South America, 14.1% from Mexico, Central America and the Caribbean, and 0.2% from Australia and the Pacific Islands. The monthly average volume of international arrivals from dengue-affected areas for the third quarter was 228,000 for the UK; 213,000 for France; 110,000 for Germany; 80,000 for Italy; 42,000 for the Netherlands; 43,000 for Spain, and 19,000 for Sweden. At the peak of the *Ae. albopictus* mosquito season for Southern Europe, which are the summer months [12]–[14], traveller arrivals from dengue-affected areas remained high (figure 4), representing around 10% of the overall number of air travellers coming into Europe. We mapped the final European destinations and corresponding volumes of global air travellers arriving from areas with dengue activity during 2010 along with the European spatial extent of *Ae. albopictus* (figure 5). The biggest increase in traveller volume was seen in the third quarter when vector populations are at the peak. Of the 442 airports in Europe, 42 were within the area of *Ae. albopictus* activity in 2010, and 36 of these airports received travellers from dengue-affected areas. The maps also display areas where *Ae. albopictus* is present and thus DENV transmission could occur. Accordingly, southern European areas are at greatest risk for autochthonous dengue outbreaks. While the presence of the vector is a necessary but not sufficient prerequisite for DENV onward transmission, a number of factors such as vector density, human exposure, access to care, etc. are also important in determining the risk of autochthonous transmission.

In 2010, a total of 703,396 travellers arrived from dengue-affected areas to airports where *Ae. albopictus* has been recorded. Two such Italian cities (Milan and Rome) received over half, and Barcelona 14% of these travellers that enter Europe from dengue-active/affected areas (table 1). All the other cities received a much smaller fraction of travellers. Compared to 2010, by 2013, the range of *Ae. albopictus* had expanded to include more European cities (with airports) which is reflected in a geographic widening of the distribution of airports at risk (table 1). Using 2010 IATA data (2013 data were not yet available), airports in the expanded area are projected to serve 978,831 travellers arriving from dengue-affected areas (Supplemental Material: Figure S1). In other words, 39% more travellers in 2013 than in 2010 were projected to arrive into those parts of Europe where *Ae. albopictus* has been introduced. This 2013 expansion is composed of areas where *Ae. albopictus* is firmly established but also areas where it was recently introduced, which corresponds to an 15% increase in projected traveller volume.

**Table 1:** Distribution of travellers from dengue affected areas (endemic and epidemic) coming into airports in the parts of Europe in 2010 where *Ae. albopictus* has been recorded in 2010 and 2013.

Selection of EU airports based on *Aedes albopictus* distribution in 2010				
Country	City airport(s)	Status of *Aedes albopictus*	Number of incoming travellers	Percentage of the overall number of passengers coming from dengue active areas
Italy	Milan	Established	187353	**26.6**
Italy	Rome	Established	186495	**26.5**
Spain	Barcelona	Established	95596	**13.6**
Italy	Venice	Established	41332	**5.9**
France	Nice	Established	31051	**4.4**
France	Marseille	Established	26103	**3.7**
Italy	Bologna	Established	24152	**3.4**
France	Ajaccio	Established	13102	**1.9**
France	Bastia	Established	11901	**1.7**
Italy	Florence	Established	9955	**1.4**
Italy	Turin	Established	8580	**1.2**

Selection of EU airports based on *Aedes albopictus* distribution in 2013				
Country	City airport(s)	Status of *Aedes albopictus*	Number of incoming travellers	Percentage of the overall number of passengers coming from dengue active areas
Italy	Milan	Established	187353	**19.2**
Italy	Rome	Established	186495	**19.1**
Spain	Barcelona	Established	95596	**9.8**
Switzerland	Geneva	Recently introduced	78950	**8.1**
Greece	Athens	Established	51635	**5.3**
Italy	Venice	Established	41332	**4.2**
France	Lyon	Established	33169	**3.4**
France	Nice	Established	31051	**3.2**
France	Toulouse	Established	27107	**2.8**
France	Marseille	Established	26103	**2.7**
Italy	Bologna	Established	24152	**2.5**
France	Bordeaux	Recently introduced	17916	**1.8**
France	Ajaccio	Established	13102	**1.3**
Switzerland	Basel/Mulhouse	Recently introduced	12876	**1.3**
France	Bastia	Established	11901	**1.2**
Italy	Florence	Established	9955	**1.0**
France	Montpellier	Established	9738	**1.0**
Italy	Turin	Established	8580	**0.9**
France	Figari	Established	8553	**0.9**

We assessed the relationship between monthly in-coming traveller volume from dengue-affected areas for 2010 and the count of dengue importations at the country level. Table 2 presents the results of the hierarchical multivariate model with estimated coefficients and incidence rate ratios (IRR), p-values and 95% confidence intervals for each variable. The number of officially notified imported dengue cases was significantly associated with the monthly number of travellers originating from dengue-affected areas. The adjusted incidence rate ratio for imported dengue cases in 2010 was 1.09 with a 95% confidence interval (95%CI) of [1.01–1.17] for every 10,000 traveller increase, which corresponds to a 9% increase in the incidence of imported cases for every additional 10,000 travellers arriving from dengue-affected areas, all other predictors in the model being constant. In August, September, and October the rate ratio was 1.70 (95%CI: 1.23–2.35), 1.46 (95%CI: 1.02–2.1), and 1.35 (95%CI: 1.01–1.81), respectively. After removing 6 outliers (Pearson residual >2.5 or <−2.5), the model coefficients remained stable. The model fit was adequate with predicted count value predicting the observed value with no evidence of model misspecification. A scatter plot of the number of observed cases and the number of model predicted counts with an R^2^ value of 0.961 is presented in Supplemental Material (Figure S2).

**Table 2:** Multilevel model for the estimation of the risk of dengue importation into Europe, by month, 2010.

Variables	Observed values	Coefficient	Confidence Interval (95%)	Incidence Rate Ratio	Confidence Interval (95%)	P-Value
Number of passenger dengue affected areas (per 10 000)		0.09	[0.01–0.16]	1.11	[1.02–1.21]	**0.021**
Month						
January*	108					
February	102	−0.18	[−0.46–0.09]	0.83	[0.63–1.1]	0.194
March	134	0.14	[−0.12–0.39]	1.15	[0.89–1.48]	0.285
April	107	−0.14	[−0.42–0.13]	0.87	[0.66–1.14]	0.303
May	131	0.03	[−0.24–0.31]	1.03	[0.79–1.36]	0.81
June	108	−0.17	[−0.45–0.12]	0.85	[0.64–1.12]	0.247
July	122	−0.32	[−0.67–0.02]	0.72	[0.51–1.02]	0.068
August	277	0.53	[0.21–0.85]	1.7	[1.23–2.35]	**0.001**
September	269	0.38	[0.02–0.74]	1.46	[1.02–2.1]	**0.04**
October	199	0.3	[0.01–0.59]	1.35	[1.01–1.81]	**0.042**
November	174	0.16	[−0.14–0.46]	1.17	[0.87–1.58]	0.302
December	127	0.06	[−0.2–0.32]	1.06	[0.82–1.37]	0.662

*: reference category for categorical variable. Number of observation n = 84. Countries was set as hierachical level (France, Germany, Italy, Spain, Sweden, Netherlands and United Kingdom) using a random-intercept and random-coefficient Negative Binomial model.

## Discussion

The extent and global reach of the contemporary air transport network has been linked to the rapid dispersal of dengue worldwide [18], [35]–[37]. Such global networks have not only facilitated the spread of the four dengue viruses (DENV 1–4) and its vectors *Ae. aegypti* and *Ae. albopictus* in tropical and subtropical areas, but it has also been principally responsible for the importation of dengue cases into other parts of the world, including Europe [20], [21], [38]. We have developed an empirical model for 2010 to assess the relationships between the number of monthly in-coming travellers and the number of monthly dengue importations at the country level. Based on the high spatial and temporal resolution of our international air traffic data, we delineate the main driver of dengue importation and its pattern into EU countries. The model accounts for dengue seasonality in the origin countries since dengue presence was recorded by month. No country-specific dengue incidence rates were incorporated in the model since such estimates can very difficult to compute on a global scale [6]. Rather, our empirical model is based on epidemic intelligence data and yields excellent quantitative correspondence between arriving travellers from dengue affected areas and the number of imported dengue cases. The importation risk for 2010 was the highest between August and October.

The importation of a viremic dengue case or infected mosquito is a necessary but not sufficient cause in the emergence and dissemination of the disease. The virus also has to be introduced into an area that is hospitable to the appropriate mosquito vector. Suitable habitats for *Ae. aegypti* and *Ae. albopictus* are defined by both environmental and climatic determinants and certain areas in Europe certainly fulfil these criteria, such as the Mediterranean [22]. Moreover, the virus has to be introduced into an area where the vector is present in sufficiently high numbers to sustain an outbreak. As the recent autochthonous transmissions in Europe have shown, this prerequisite can also be met at certain times of the year [13], [20], [21].

The emergence and spread of dengue are also determined by other factors such as vector-exposure patterns in the population, access to care, etc. However, we did not model the subsequent autochthonous transmission as it has been done for other diseases [39]. Rather, our model for 2010 quantifies the likelihood and timing of importation. Since no highly efficacious dengue vaccine is currently available [7], mounting the most appropriate and targeted public health response is crucial. In light of budget shortfalls in public health due to the economic crisis [40], the model presented here can be applied to other settings and timeframes in order to restrict the spatiotemporal extent of these interventions to high risk areas for cost-effectiveness reasons with local and regional surveillance. For example, seasonal or sentinel surveillance can be enhanced and tailored to certain regions and time periods. Such models can also help to pragmatically schedule vector surveillance (vector presence, absence, recent introduction, and density) as well as vector abatement activities (e.g. destruction of breeding sites) to the most crucial time period and at-risk areas. In addition, public information campaigns and education of health care providers can be refined with the help of such models to increase awareness regarding the (re-) emergence of this tropical disease in Europe. This approach could be useful for both prospective travellers (e.g. exposure prevention) and health care providers of returning travellers (treatment).

The approach presented in this study could also be adapted to other tropical diseases, such as Chikungunya fever, and to other regions of the world [41], [42] to help define areas and timing of enhanced surveillance and rapid response (including active case finding and contact tracing) during the season of high mosquito activity [13]. Other control measures for vector-borne diseases exist at airports but might be unrealistic to implement in Europe: vectors concealed in aircrafts can be exterminated through disinfection of planes by pesticide spraying; exposure of infected individuals to competent vectors can be reduced through isolation and containment; and importation of viremic patients can be intercepted through airport screening of symptomatic travellers. However, all of these strategies have considerable shortcomings. Thus, these quantitative models can chart a pragmatic course of action.

### Limitations

Dengue cases have been reported to ECDC since 2008, the bulk of cases being imported from Asia [30]. The majority of countries have a passive, but compulsory surveillance system. Nevertheless, several countries never report dengue data to ECDC [43]. Moreover, there is also a need to implement a more specific case-definition for surveillance purposes on a European level. Based on these constraints it is likely that the European surveillance system for dengue is not very sensitive to pick up symptomatic cases, let alone asymptomatic and/or sub/clinical infections. Quantitative models such as our 2010 model can help assess the risk for dengue importation and spread in the absence of sensitive surveillance data. However, propagation of disease spread is a function not only of disease importations but also of the number of secondary cases generated by imported cases in a susceptible population (reproduction number) and the average time for secondary cases to occur. The model presented in this study does not factor in these location-specific parameters, as they are not known. Additionally, the model does not account for stopovers or length of stopovers that have been previously described [18], [35]. Nevertheless, it can quantify the statistically significant relationships between the number of travellers and local imported cases.

The independent variable of imported cases might have been underestimated if visitors from dengue areas were not able to access health care in the destination countries. Moreover, the accuracy of the 2010 model also depends on the sensitivity of dengue notifications internationally. To assure high data accuracy, data were compiled from complementary and overlapping sources such as the weekly compilation of the dengue assessment by epidemic intelligence at ECDC and published in the CDTR; we also extracted data from HealthMap, MediSys, and PubMed and crosschecked with CDTR information.

The selection of a “traveller catchment area” of 200 km radius around airports for dengue presence was chosen based on the assumption that a person will travel a maximum of 2 hours to arrive at their nearest airport location, at an average speed of 80–100 km/hr on main highways [44], [45]. The 2 hour travel time only considers road transportation, so additional travel distance was added for people arriving at major airports through high speed rail systems [45]. The size of this radius was compared against the size of international ground transportation networks (roads, trains, etc.); we considered buffers of 150, 200 and 250 km but selected 200 km based on the size of these networks. In practice, it is likely that actual “traveller catchment areas” vary by country or region, due to varying socioeconomic contexts, infrastructures (e.g. rapid rail links to airports, or not), and so on [46].

### Conclusion

Our model for 2010 suggests that the risk of dengue importation into Europe is greatest in August, September and October. The 2010 model presented here identifies three large European cities (Milan, Rome, and Barcelona) with vector presence as disproportionally affected by travellers arriving from dengue-active areas. Due to the geographic expansion of *Aedes* mosquitoes from 2010 to 2013 into novel areas with airport services, 39% more travellers are projected to arrive from dengue-affected areas into Europe where the vector is now present.

International dispersal of dengue will increasingly take advantage of an expanding global transportation network and will no longer be hampered by protracted travel times (>viremic period). The recent transformation of traditional disease dispersal patterns through air traffic is the inevitable consequence of globalization; thus pathogen introduction is difficult to intercept and public health has to rely on early detection, rapid response and effective control measures in order to contain potential dengue establishment and spread [47]. At a time when the dengue community realizes that the current control approaches are too simplistic, quantitative models presents a novel tool in the arsenal against dengue [48]. Such empirical models lend themselves to mount more effective public health responses and can be developed into early warning systems of emerging risks [49], [50].

## Supporting Information

Figure S1 Projected airport-level final destination of international travellers from dengue affected areas by quarter for 2010, overlaid with the presence of *Ae. albopictus*, 2013.
(PDF)

Figure S2 Scatter plot between numbers of imported cases observed and predicted count by the model.
(PDF)
