# Supplementary figures and images for: International Dispersal of Dengue through Air Travel: Importation Risk for Europe
Source: PLoS Negl Trop Dis. 2014 Dec 4;8(12):e3278. doi: 10.1371/journal.pntd.0003278 (PMC4256202; doi:10.1371/journal.pntd.0003278)

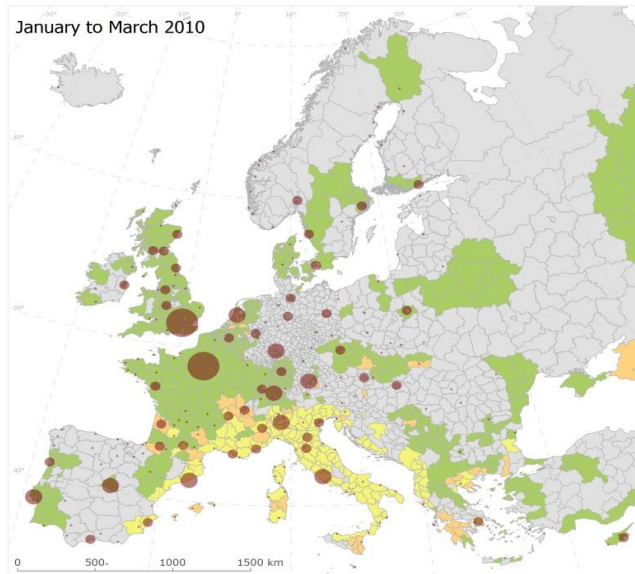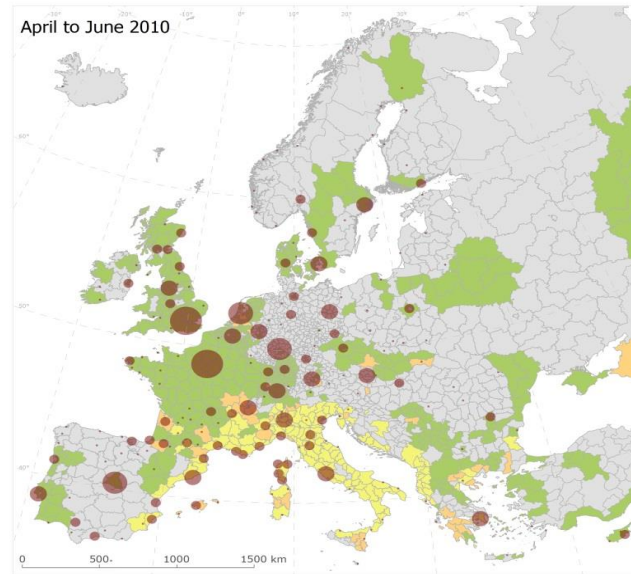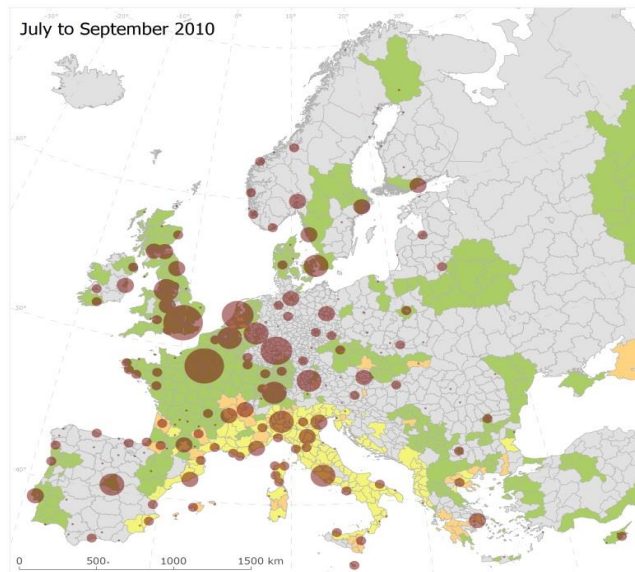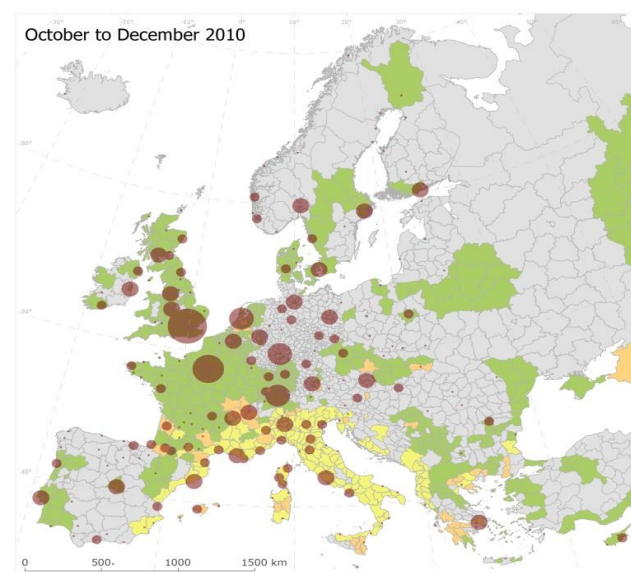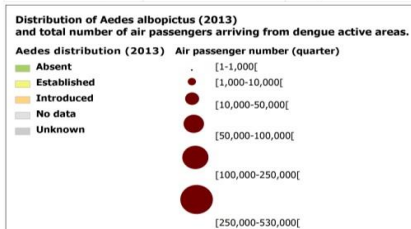

Supplement: Figure S1 — Projected airport-level final destination of international travellers from dengue affected areas by quarter for 2010, overlaid with the presence of Ae. albopictus, 2013. (PDF) [file pntd.0003278.s001.pdf]

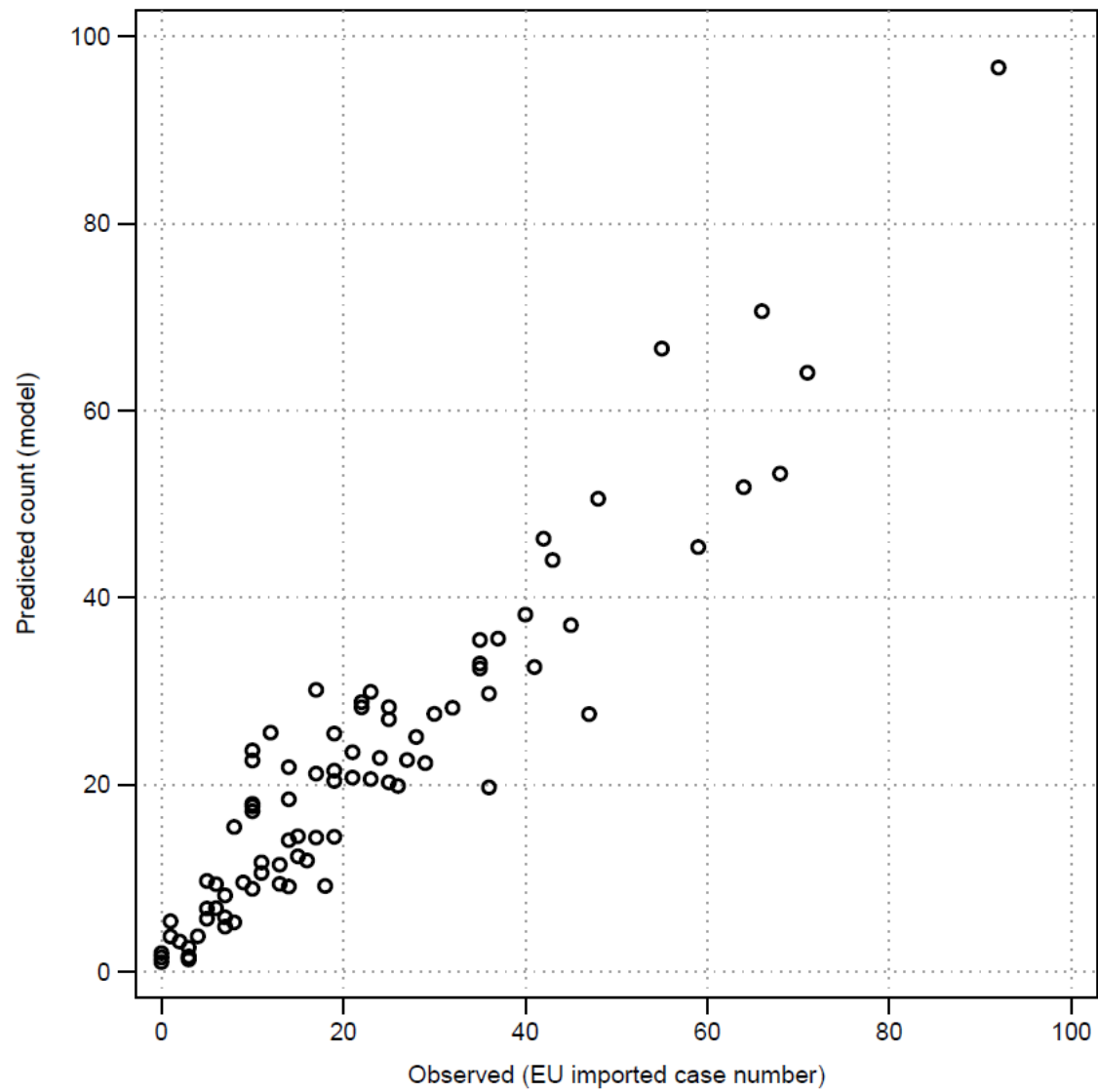

Supplement: Figure S2 — Scatter plot between numbers of imported cases observed and predicted count by the model. (PDF) [file pntd.0003278.s002.pdf]
